# The Controversies of Coronary Artery Disease in End-Stage Kidney Disease Patients: A Narrative Review

**DOI:** 10.31083/j.rcm2406181

**Published:** 2023-06-25

**Authors:** Daniel Hirsch, Brandon Lau, Virag Kushwaha, Kenneth Yong

**Affiliations:** ^1^Department of Nephrology, Prince of Wales Hospital, Randwick, NSW 2031, Australia; ^2^Prince of Wales Clinical School, University of New South Wales, Kensington, NSW 2033, Australia; ^3^Department of Cardiology, Prince of Wales Hospital, Randwick, NSW 2031, Australia

**Keywords:** coronary artery disease, end-stage kidney disease, atherosclerosis, dialysis modality, chronic inflammation, coronary artery disease screening, revascularisation

## Abstract

Cardiovascular disease (CVD) accounts for more than 50% of deaths among patients with end-stage kidney disease (ESKD). Approximately 40–50% of ESKD patients have clinically significant coronary artery disease (CAD) due to atherosclerosis which accounts for a significant proportion of CVD risk. However, other CVD pathologies including myocardial fibrosis, vascular calcification and arterial stiffening play important contributory roles. The pathophysiology of CAD in ESKD is distinct from the general population. ESKD patients is typically have diffuse multi-vessel involvement with increased calcification that involves both intimal and medial layers of the arterial wall. There is a complex interplay between an increased burden of traditional Framingham risk factors and exposure to non-traditional risk factors including chronic inflammation and dialysis *per se*. Established treatments for CAD risk factors including cholesterol lowering with statin therapy have attenuated effects and ESKD patients also have worse outcomes after revascularisation. Recent trials such as the Canakinumab Anti-Inflammatory Thrombosis Outcomes Study (CANTOS) have established that direct modulation of inflammation improves CVD outcomes in the general population, which may prove to be a potential attractive therapeutic target in ESKD patients. Multiple retrospective observational studies comparing mortality outcomes between haemodialysis (HD) and peritoneal dialysis (PD) patients have been inconclusive. Randomised trials on this issue of clinical equipoise are clearly warranted but are unlikely to be feasible. Screening for stable CAD in asymptomatic ESKD patients remains a clinical dilemma which is unique to chronic dialysis patients being assessed for kidney transplantation. This has become particularly relevant in light of the recent ISCHEMIA-CKD trial which demonstrated no difference between optimal medical therapy and revascularisation upon CVD outcomes or mortality. The optimal strategy for screening is currently being investigated in the ongoing large international multi-centre CARSK trial. Here we discuss the pathophysiology, risk modification, treatment, screening and future directions of CAD in ESKD.

## 1. Introduction

Chronic kidney disease (CKD) is defined as reduced glomerular filtration rate (GFR; <60 mL/min/1.73 $m^{2}$) or elevated albuminuria for at least 3 months [1, 2]. Measurement of estimated GFR (eGFR) from the Modification of Diet in Renal Disease (MDRD) or CKD Epidemiology Collaboration (CKDEPI) equations has become an important epidemiological cornerstone for identifying and staging of CKD (Table 1, Ref. [2]; Table 2, Ref. [2]) [3, 4]. Stage 5 CKD includes end-stage kidney disease (ESKD) patients receiving renal replacement therapy (dialysis or transplant).

**Table 1.** **Chronic kidney disease staging [2]**.

CKD Stage	GFR (mL/min/1.73 $m^{2}$)	Terms
Stage 1	≥90	Normal/high
Stage 2	60–89	Mild decrease*
Stage 3a	45–59	Mild to moderate decrease
Stage 3b	30–44	Moderate to severe decrease
Stage 4	15–29	Severe decrease
Stage 5	<15	Kidney failure (ESKD)

CKD, chronic kidney disease; GFR, glomerular filtration rate; ESKD, end-stage kidney disease. *relative to healthy young adults. Stages 1 and 2 alone do not fulfil criteria for CKD alone in the absence of other markers of kidney damage (albuminuria).

**Table 2.** **Albuminuria categories & CKD [2]**.

Albuminuria Category	AER (mg/24 hrs)	ACR (mg/mmol)	ACR (mg/g)	Terms
A1	<30	<3	<30	Normal to mild
A2	30–300	3–30	30–300	Moderate
A3	>300	>30	>300	Severe*

CKD, chronic kidney disease; AER, albumin excretion ratio; ACR, albumin to creatinine ratio. *includes nephrotic syndrome (AER >2200 mg/24 hrs or ACR >220 mg/mmol; >2200 mg/g).

CKD has an estimated prevalence of 10–15% worldwide and represents a major public health problem because of its strong association with adverse outcomes, most notably increased all-cause mortality risk [5, 6, 7, 8, 9, 10, 11, 12]. The 2020 Global Disease Burden study ranked CKD amongst the top 10 contributors to poor prognosis globally. CKD rose from the 27th (15.7 deaths per 100,000 people) to the 11th (18.2 deaths per 100,000 people) leading cause of death worldwide between 1990 and 2016. This represents the 3rd largest increase behind dementia and human immunodeficiency virus/acquired immunodeficiency syndrome [13, 14].

**Fig. 1.**
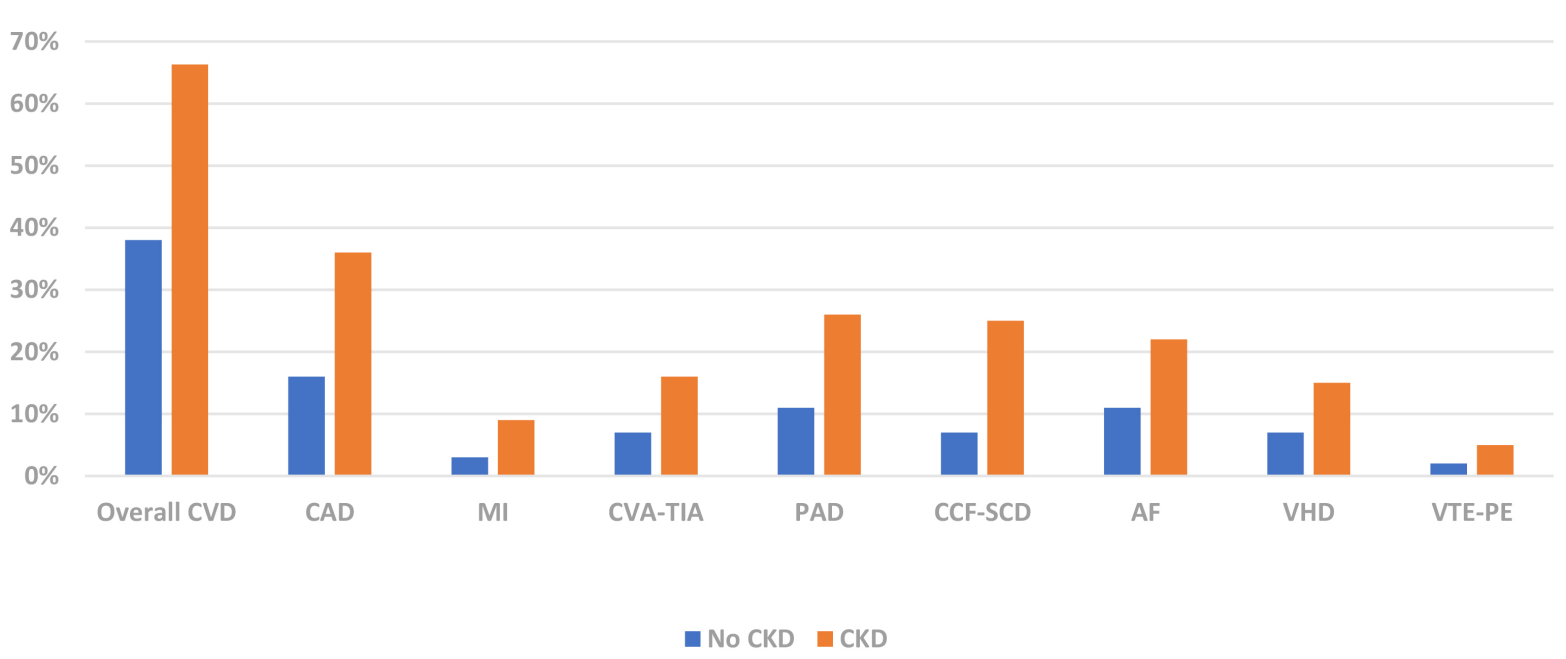
**CVD Phenotype Prevalence by CKD Status (USRDS 2020) [21]**. CVD, cardiovascular disease; CAD, coronary artery disease; MI, myocardial infarct; CVA-TIA, cerebrovascular accident-transient ischaemic attack; PAD, peripheral arterial disease; CCF-SCD, congestive cardiac failure-sudden cardiac death; AF, atrial fibrillation; VHD, valvular heart disease; VTE-PE, venous thromboembolism-pulmonary embolism; CKD, chronic kidney disease; USRDS, United States Renal Data Services.

Kidney and heart pathologies share a close and inter-dependent relationship [15, 16, 17]. The presence of cardiovascular disease (CVD) is associated with progression of CKD and vice versa. A prime example of this is the cardiorenal syndrome which is a multi-system disorder characterised by simultaneous heart and kidney dysfunction as a result of complex metabolic, haemodynamic, neurohormonal and inflammatory pathways [18, 19, 20]. CKD is strongly associated with a variety of severe CVD phenotypes and CKD patients have a significantly higher burden of both atherosclerotic and non-atherosclerotic forms of CVD compared to those without CKD (Fig. 1) [21]. This disproportionate burden of CVD is most evident in younger patient groups. The 2020 United States Renal Data Services (USRDS) annual report demonstrated a higher prevalence of CAD in CKD patients aged 66–69 years compared to those aged ≥85 years but without CKD. This phenomenon of accelerated ‘vascular ageing’ is specific to CKD and highlights the importance of CAD as a leading cause of CVD in CKD.

In 2013, 4% of deaths (2.2 million) worldwide were attributable to CKD and more than 50% of these (1.2 million) were due to cardiovascular disease (CVD) [22]. CVD is the leading cause of mortality in CKD patients and is often regarded as the true burden of CKD. The risk of mortality progressively rises in parallel with increasing severity of CKD [10, 11, 12]. The CKD Prognosis Consortium has demonstrated that the probability of developing coronary artery disease (CAD) increases linearly below a GFR threshold less than 60 mL/min/1.73 $m^{2}$ [23]. CVD mortality is 5–10 fold higher in individuals with pre-dialysis CKD and 30–100 fold in ESKD patients compared to the age- and gender-matched general population [10, 12, 24]. This makes ESKD patients one of the highest risk groups for CVD.

While extensively studied in pre-dialysis CKD, there remains a paucity of data on CAD in the ESKD population. This review discusses the unique pathophysiology, clinical presentations and dilemmas of CAD in ESKD with specific focus upon chronic dialysis patients, including screening and management of asymptomatic disease in stable patients on the transplant wait-list.

## 2. Pathophysiology: Arterial Disease & CKD

CKD is associated with profound structural changes to the heart and blood vessels. Chronic exposure to the uraemic milieu, dialysis procedure *per se *(in the case of ESKD patients) and heavy prevalence of traditional CVD risk factors appears to promote accelerated vascular ageing, evidenced by biological age markers such as telomere shortening [17, 25].

**Fig. 2.**
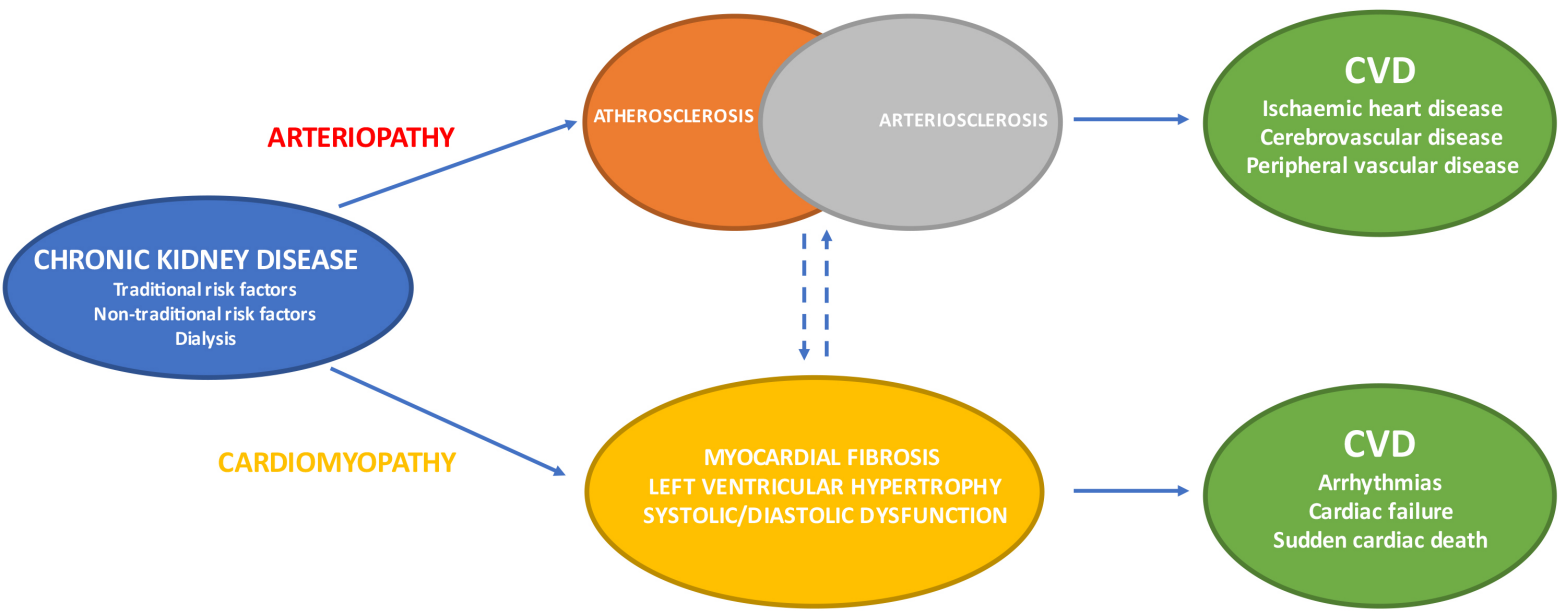
**CKD: arterial & cardiac disease resulting in varied CVD phenotypes**. CVD, cardiovascular disease; CKD, chronic kidney disease.

Arterial disease in CKD can be classified as atherosclerosis and arteriosclerosis (also known as Monckeberg’s sclerosis) (Fig. 2) [26, 27, 28, 29, 30]. The former is an intimal disease which is characterised by the formation of intimal atheromatous plaques causing occlusive narrowing of the arterial lumen. The latter is a chronic non-occlusive disease which results in large artery dilatation, medial hypertrophy and arterial calcifications. Atherosclerosis negatively impacts arterial conduit function resulting in reduced tissue perfusion and organ ischaemia. Atherosclerosis is the central feature of CAD with acute plaque rupture/thrombosis and chronic occlusive disease manifesting clinically as acute coronary syndromes (ACS) and stable angina respectively. Arteriosclerosis is associated with reduced arterial compliance, widened pulse pressures and increased vascular stiffness which have deleterious end-organ effects upon the kidney, heart and brain. The cardiac effects of increased arterial stiffness include decreased coronary perfusion, subendocardial ischaemia, myocardial fibrosis, diastolic dysfunction and left ventricular hypertrophy (LVH).

The widespread effects of uraemia upon the cardiovascular system generates large variation in CVD phenotypes observed in CKD and ESKD. The prevalence of sudden death, arrhythmias and cardiac failure become disproportionately elevated as kidney function declines and are amongst the leading causes of mortality in ESKD patients (Fig. 1) [17, 21]. Moreover, the negative impact of impaired kidney function upon outcomes in patients with ACS has been well described [31]. Acute kidney injury is present in approximately 25% of patients with ACS and is an independent predictor of in-hospital mortality [32]. In a post-hoc analysis of the Valsartan in Acute Myocardial Infarction Trial (VALIANT), every reduction in eGFR of 10 mL/min/1.73 $m^{2}$ was associated with an increased hazard ratio of 1.10 for death and non-fatal cardiovascular outcomes [33].

Some but not all of this increase could be explained by CAD and suggests the importance of other underlying disease processes such as myocardial fibrosis. Large-randomised trials such as the Deutsche Diabetes Dialyse (4D) study observed that only 9% of overall mortality in chronic dialysis patients was attributable to CAD compared to 26% and 7% due to sudden cardiac death and cardiac failure respectively [34]. Nevertheless, CAD remains an important cause of CVD-related morbidity and mortality in ESKD patients.

## 3. CAD & ESKD

### 3.1 Dialysis

CAD due to atherosclerosis is highly prevalent in the chronic dialysis population [35, 36, 37]. However, the true incidence and prevalence of CAD in ESKD patients is unclear. Approximately one-third of ESKD patients have an established diagnosis of CAD at the time of dialysis initiation. Angiographic studies have identified significant coronary disease (≥50% stenosis) in up to 40–50% of symptomatic and asymptomatic dialysis patients. Moreover, CAD in ESKD patients is characterised by diffuse complex disease with multi-vessel involvement (Table 3) [38, 39, 40, 41, 42].

**Table 3.** **Angiographic studies & CAD prevalence in ESKD patients [38, 39, 40, 41, 42]**.

Study	Population	Findings
Rostand 1984 [38]	Prevalent HD with angina/MI (n = 44)	53% with significant disease (>50% stenosis)
Joki 1997 [39]	Incident asymptomatic HD (n = 24)	63% with significant disease (>75% stenosis)
		74% with multivessel disease
		30–50% diffuse complex lesions
Ohtake 2005 [40]	Incident asymptomatic HD/PD (n = 30)	53% with significant disease (>50% stenosis)
Charytan 2007 [41]	Prevalent asymptomatic HD (n = 67)	42% with significant disease (>50% stenosis)
		75% with multivessel disease
Atkinson 2011 [42]	Prevalent asymptomatic HD/PD undergoing pre-transplant workup (n = 47)	47% with significant disease (>50% stenosis)

HD, haemodialysis; PD, peritoneal dialysis; MI, myocardial infarction; CAD, coronary artery disease; ESKD, end-stage kidney disease.

**Fig. 3.**
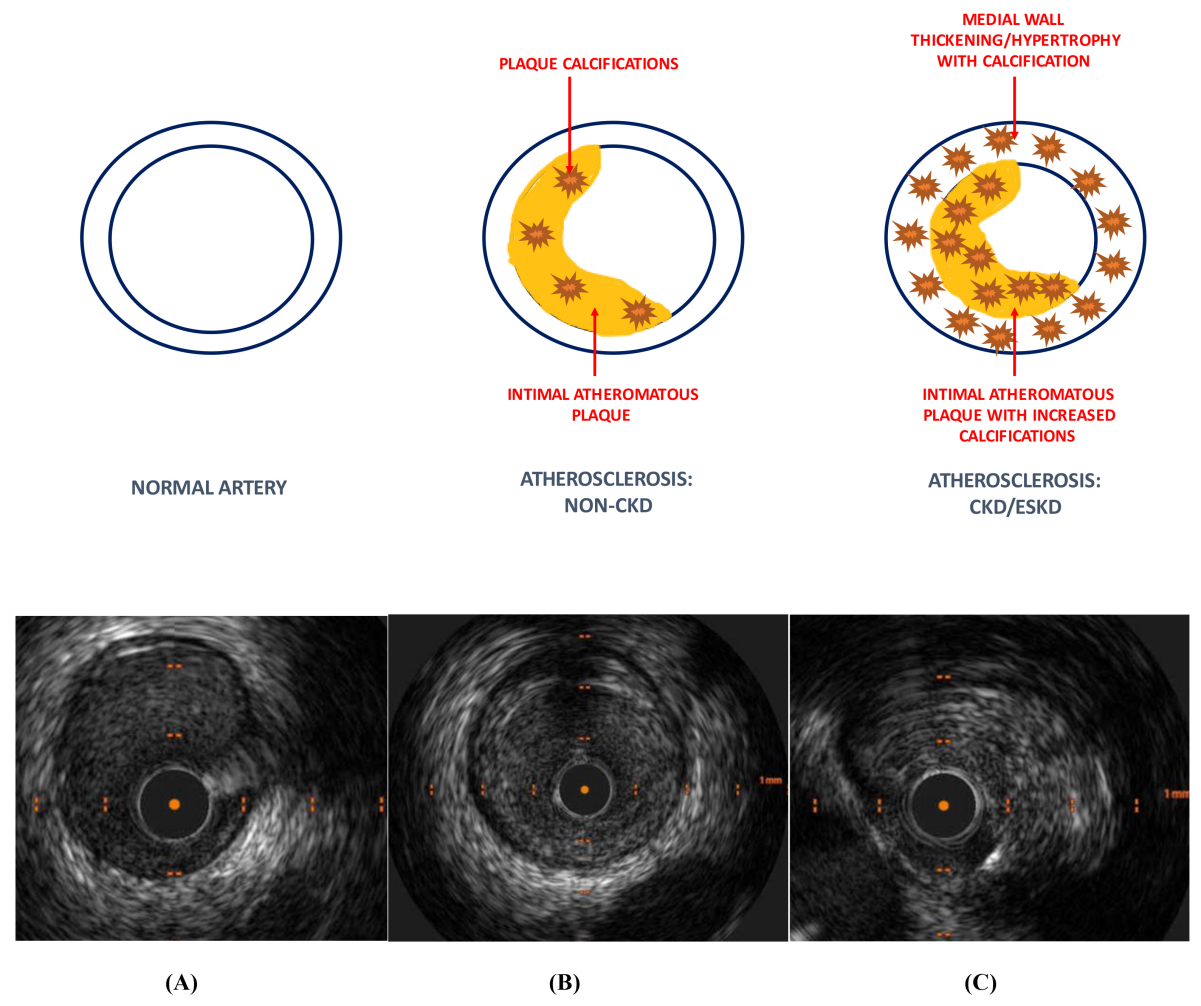
**Coronary artery disease morphology (non-CKD vs CKD/ESKD)**. Coronary artery disease in the non-CKD population is predominantly an intimal disease with some intra-plaque calcification. CKD/ESKD patients have increased plaque calcification with thickening/hypertrophy and calcification of the arterial medial wall compared to non-CKD patients. IVUS images depicting (A) normal coronary artery, (B) atherosclerosis: non-CKD and (C) atherosclerosis: CKD/ESKD. Higher burden of plaque and deep calcification in ESKD patients within the intimal and medial layers. CKD, chronic kidney disease; ESKD, end-stage kidney disease; IVUS, intravascular ultrasound.

Autopsy studies have demonstrated qualitative rather than quantitative differences in CAD due to the presence of CKD [37, 43]. CKD patients appear to have similar amount of atheromatous plaque compared to non-CKD patients. However, coronary plaques in advanced CKD and ESKD patients are typically characterised by hydroxy-apatite deposition causing increased intimal and medial calcification as well as thickening of the arterial medial wall [44, 45]. This contrasts with coronary lesions in non-CKD patients in whom atheroma plaque and calcifications are mainly limited to the intimal layer alone (Fig. 3). These differences in plaque morphology result in significantly higher coronary artery calcium (CAC) scores in CKD patients compared to those without CKD [46, 47, 48]. Arterial media calcifications have also been shown to be strong independent predictors of all-cause and CVD mortality in chronic dialysis patients [49].

Additionally, evidence suggest that uraemia has important effects upon distal coronary arteriolar beds (small vessel disease). ESKD patients have reduced myocardial capillary length density (capillary length per unit myocardial volume), increased myocyte diameter and expanded myocardial interstitial volume compared to healthy individuals and hypertensive patients with normal kidney function [50]. This ‘myocyte-capillary mismatch’ is implicated in the development of cardiomyocyte hypertrophy, myocardial interstitial fibrosis and LVH in ESKD. Coronary microcirculatory dysfunction is a likely contributor to the high false positivity rates seen with non-invasive stress testing as well as reduced clinical benefits from coronary revascularisation in ESKD patients [37].

CAD-related syndromes such as ST-elevation myocardial infarction (STEMI), non-ST elevation acute coronary syndromes (NSTEACS) and stable angina are exceedingly common in ESKD patients. The 2013 USRDS annual report estimated the annual prevalence rate of myocardial infarction (MI) in ESKD patients to be approximately 10% [51]. CAD is more likely to initially present as a NSTEACS than stable angina in ESKD patients [52]. Furthermore, ESKD patients are more likely to have NSTEACS than STEMI [53]. The increased presentations of NSTEACS in ESKD patients could be driven by small vessel disease (as described above), ischaemic pre-conditioning or collateralisation of blood vessels.

The typical clinical presentations of CAD are often modified by ESKD and the classical triad of ischaemic symptoms, electrocardiographic changes and elevated cardiac damage biomarkers is often absent in this population [54, 55]. ESKD patients are more likely to present with unexplained dyspnoea, reduced functional capacity, cardiac arrhythmia, recurrent fluid overload or hypotension and are often mistaken as cardiac failure. A retrospective analysis of the USRDS reported that only 44% of dialysis patients with acute MI presented with chest pain compared to 68% of non-dialysis patients. Furthermore, only 65% received a correct diagnosis of ACS compared to 79% of non-dialysis patients [55]. Identification of classical ischaemic ST-segment changes can be challenging in the presence of pre-existing LVH, the most common cardiac abnormality in ESKD patients. Cardiac troponins are often chronically elevated in ESKD patients for other reasons other than ischaemia including myocardial apoptosis, small vessel disease, LVH and reduced GFR (due to its being freely filtered by the glomerulus under physiological conditions) [35, 36, 56]. Elevated troponin levels are also independent predictors of subclinical CAD, CVD outcomes and poor survival in asymptomatic dialysis patients even in the absence of ACS [57, 58, 59, 60, 61]. The interpretation of these biomarkers of cardiac damage in the context of ACS is therefore challenging.

Clinical outcomes for chronic dialysis patients after acute coronary events remain poor. The WAVE-2 study observed extremely high 1-, 2- and 5-year mortality rates of 60%, 70% and 90% respectively for chronic dialysis patients with acute MI [62]. The modern era of medical and revascularisation therapies has shown some improvement in 1- and 2-year post-MI mortality to 28% and 47% respectively, but even these figures are far in excess to that of the general population [21].

### 3.2 Renal Transplantation

Renal transplantation is the optimal therapy for ESKD patients. Renal transplantation provides significant survival and quality of life benefits over chronic dialysis therapy. A retrospective analysis of the USRDS observed a 68% reduction in 3-year mortality in deceased donor transplant recipients compared to ESKD patients remaining on the transplant wait-list [63]. A systematic review of 110 studies with a total of 1.9 million transplant recipients found significantly lower mortality associated with transplantation with an increasing relative magnitude of benefit over time and reduced risk of CVD events [64].

**Fig. 4.**
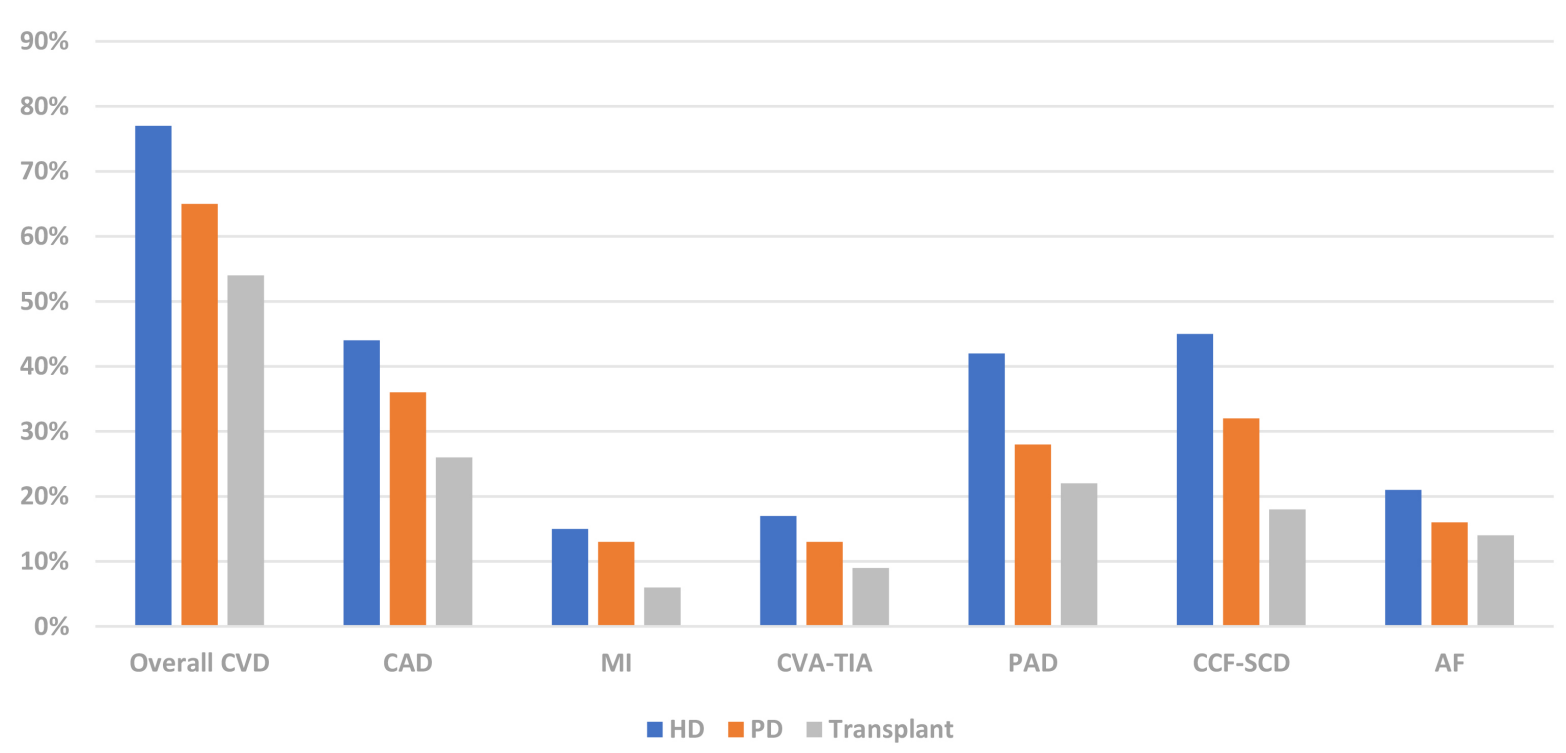
**Prevalence of CVD phenotypes in dialysis & renal transplant recipients (USRDS 2020) [21]**. CVD, cardiovascular disease; CAD, coronary artery disease; MI, myocardial infarction; CVA-TIA, cerebrovascular disease-transient ischaemic attack; PAD, peripheral arterial disease; CCF-SCD, congestive cardiac failure-sudden cardiac death; AF, atrial fibrillation; HD, haemodialysis; PD, peritoneal dialysis; USRDS, United States Renal Data Services.

**Fig. 5.**
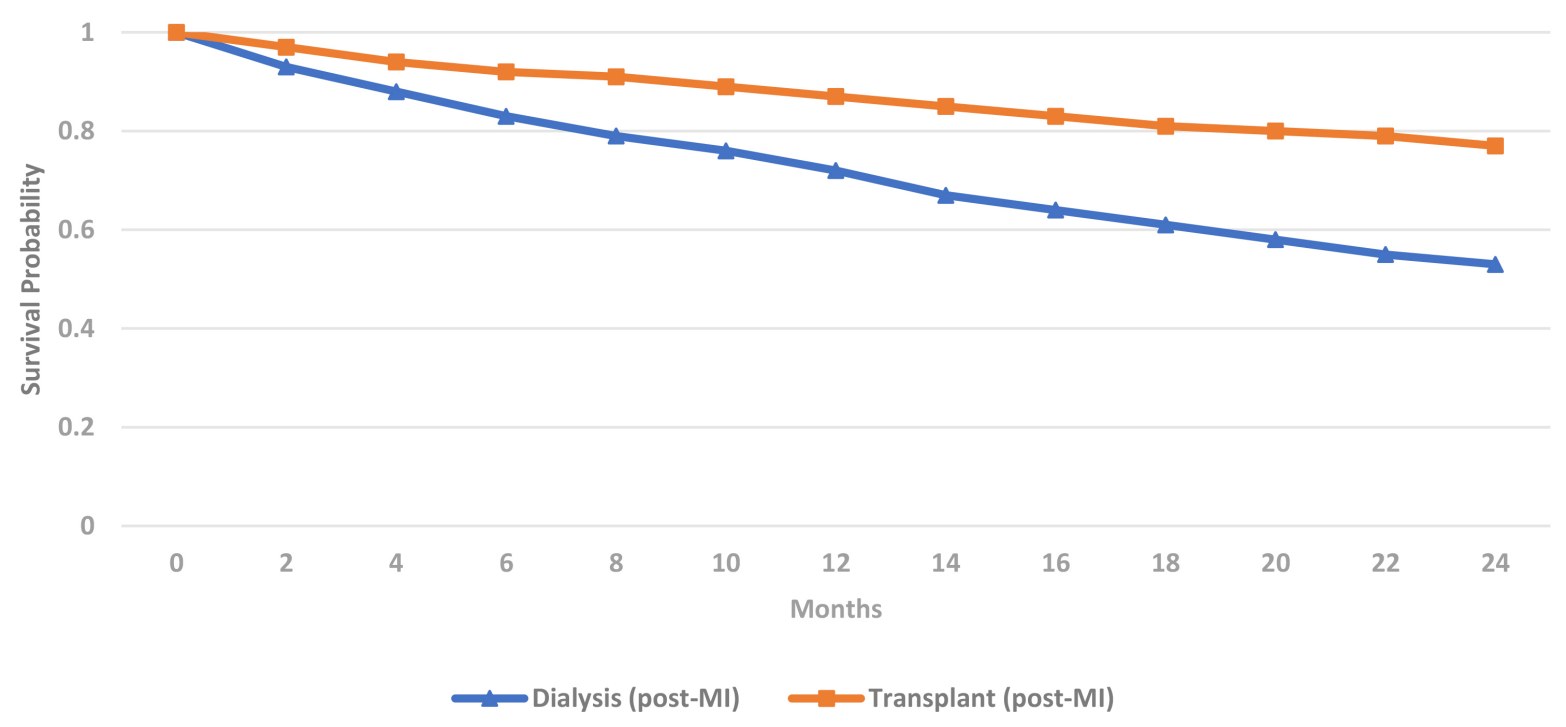
**Post-myocardial infarction adjusted survival in ESKD patients by treatment modality (USRDS 2020) [21]**. MI, myocardial infarction; USRDS, United States Renal Data Services; ESKD, end-stage kidney disease.

Renal transplant recipients have a reduced burden of CVD compared to chronic dialysis patients (Fig. 4) [21]. The survival benefit of renal transplantation is largely attributable to abrogation of CVD. The USRDS reported a lower incidence of ACS in diabetic ESKD patients after renal transplantation (0.79% per patient year) compared to the period prior to transplant (1.67% per patient year) [65]. Furthermore, renal transplantation was independently associated with a 0.38-fold decreased risk of ACS compared to wait-listed dialysis patients who never underwent transplantation. The 2020 USRDS report also observed superior 1- and 2- year survival in renal transplant recipients (87% and 77% respectively) compared to chronic dialysis patients (72% and 53% respectively) after myocardial infarction (Fig. 5) [21]. Despite inherent flaws such as selection bias, these retrospective data suggest that the correction of uraemia with renal transplantation reduces the frequency and severity of CAD.

**Fig. 6.**
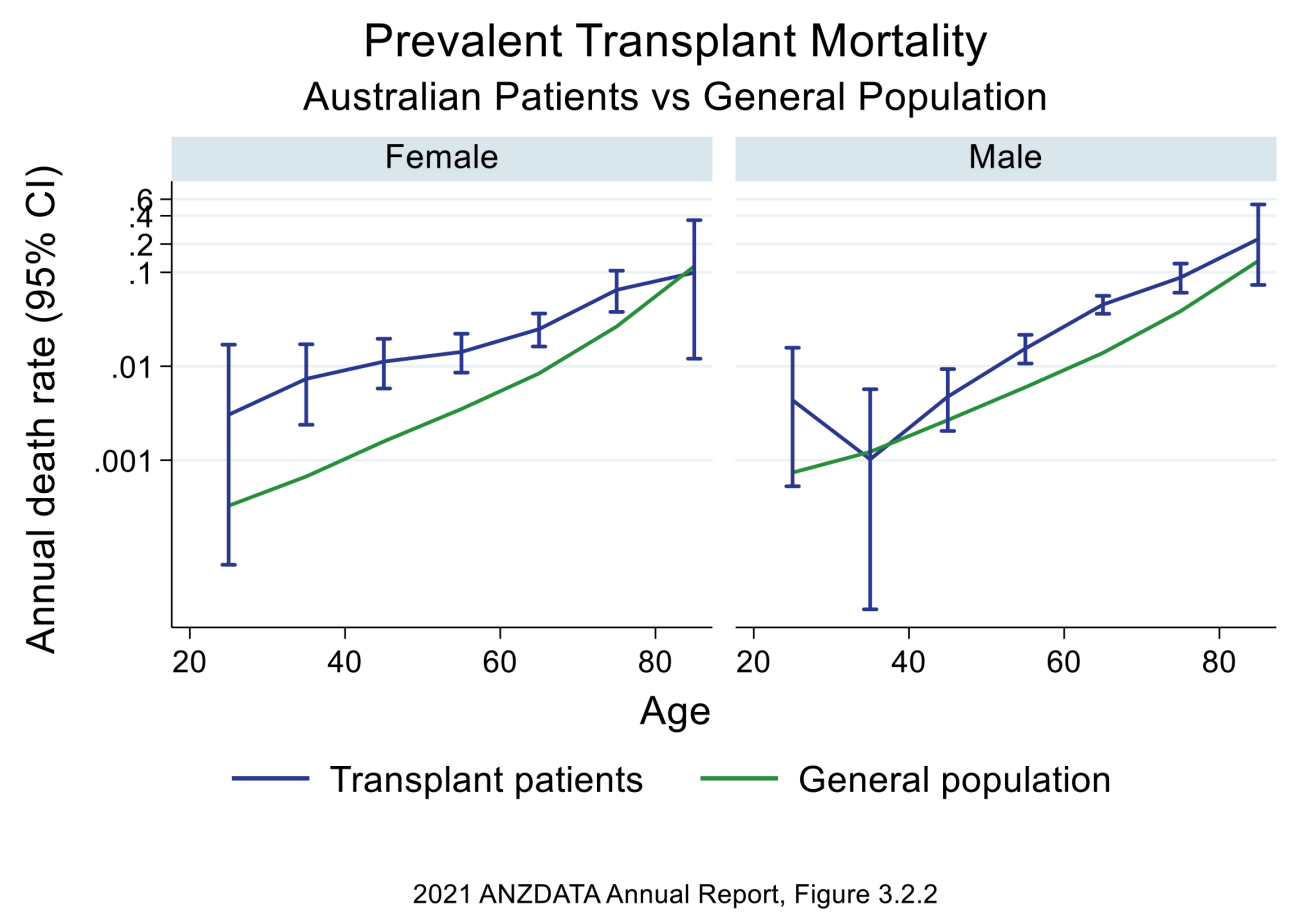
**Transplant mortality vs general population by gender: Australia (ANZDATA 2021) report [66]**. Figure used with permission from ANZDATA. ANZDATA, Australian & New Zealand Dialysis & Transplant.

**Fig. 7.**
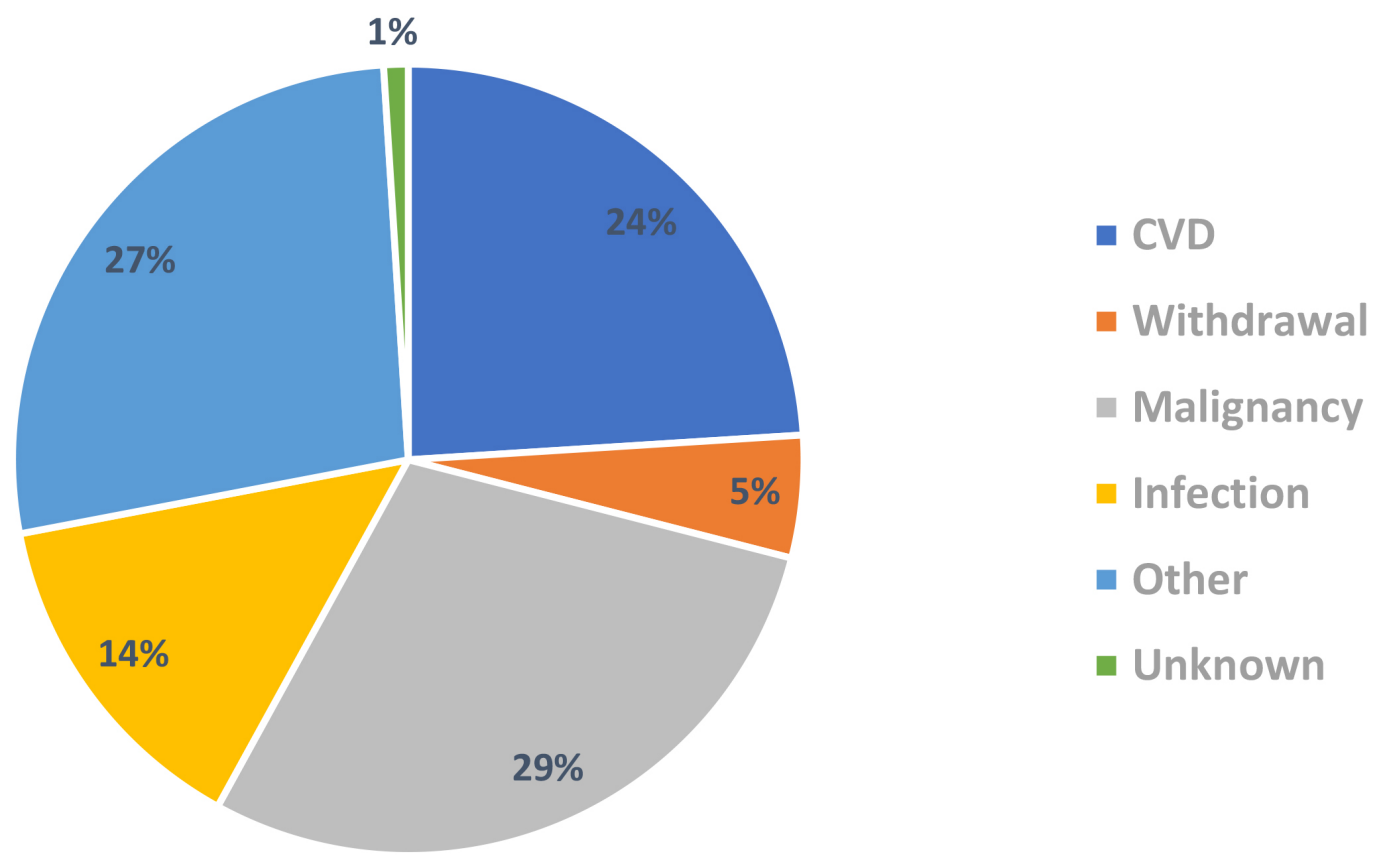
**Causes of death with functioning graft in renal transplant recipients 2016–2020 (ANZDATA 2021 report) [67]**. ANZDATA, Australian & New Zealand Dialysis & Transplant; CVD, coronary artery disease.

Renal transplant recipients still have increased mortality in comparison to the general population (Fig. 6) [66]. Death with functioning graft (48%) was the most likely outcome in renal transplant recipients reported by the Australian & New Zealand Dialysis & Transplant (ANZDATA) registry. CVD (24%) was the 3rd leading cause of death with functioning graft in Australia and New Zealand from 2016–2020 (Fig. 7) [67]. Therefore, CVD remains an important cause of mortality and morbidity in the renal transplant population even after adjusting for competing risks such as infection and malignancy.

Renal transplant recipients have a unique CAD risk profile which includes an increased prevalence of traditional risk factors (diabetes, hypertension, obesity and dyslipidaemia) and transplant-specific factors which include the cardio-metabolic adverse effects of immunosuppression, chronic inflammation and decreased graft function [68]. The management of CVD in the renal transplant recipients is not fully understood and the under-representation of CKD and/or renal transplant patients from major outcome trials remains a limiting factor in the treatment of CVD in this population.

## 4. CAD Risk Factors in ESKD

The pathogenesis of CAD in ESKD patients involves a complex interplay between traditional and non-traditional risk factors (Table 4) [16, 69, 70]. The latter includes risk factors unique to CKD/ESKD patients including chronic inflammation and dialysis *per se*.

**Table 4.** **CAD risk factors in ESKD**.

	Non-modifiable	Modifiable
Traditional	Age	Smoking
	Gender	Diabetes
	Ethnicity	Hypertension
	Family history	Dyslipidaemia
	General	Uraemia specific
Non-traditional	Oxidative stress	Dialysis
	Arterial stiffness	Anaemia
	Hypercoagulability	Albuminuria
	Vascular calcification	Uraemic toxins
	Chronic inflammation	Volume overload
	Endothelial dysfunction	Hyperparathyroidism
	Hyperhomocysteinaemia	Protein carbamylation
	Sympathetic overactivity	Protein-energy wasting
	Left ventricular hypertrophy	Abnormal mineral metabolism

CAD, coronary artery disease; ESKD, end-stage kidney disease.

### 4.1 Traditional Risk Factors

Traditional risk factors play important roles in the pathogenesis of CAD [70]. Medical therapies for atherosclerotic plaque stabilisation and revascularisation for critical lesions remain the cornerstone of CAD treatment in ESKD. In the Framingham Heart Study, participants with CKD were older and had increased prevalence of hypertension, obesity and dyslipidaemia [71]. Those with CKD were also less likely to achieve optimal blood pressures or cholesterol levels despite treatment. The National Health and Nutrition Examination Survey (NHANES) III study observed that more advanced and severe kidney dysfunction was associated with increased numbers of Framingham risk factors [72].

Traditional Framingham risk factors do not entirely explain CVD risk in CKD patients [16, 69, 70]. The Haemodialysis (HEMO) study observed that neither cholesterol levels or pre-dialysis systolic blood pressures were associated with CAD [73]. The Framingham risk tool has also been found to have poor predictive value in CKD and ESKD cohorts [74, 75]. The association between traditional Framingham risk factors and CVD is complicated by the ‘reverse epidemiology’ phenomenon in which well-established relationships between risk factors and CVD in the general population such as hypertension, dyslipidaemia and obesity do not exist or may even be reversed in the ESKD population [76]. For example, lower levels of blood pressure (systolic blood pressure <120 mmHg) are paradoxically associated with decreased survival in ESKD patients and mortality risk lowest with pre-dialysis systolic blood pressures of 160–189 mmHg [77]. Similar relationships with cholesterol (<5.0 mmol/L) and body mass index (<20 kg/$m^{2}$) have also been described in other ESKD cohorts [78, 79].

### 4.2 Non-Traditional Risk Factors

ESKD patients are exposed to a variety of non-traditional risk factors which amplify CVD risk [16, 69, 70]. Some are unique to uraemia while others such as chronic inflammation are also present in the general population (Table 4). The relative importance and specific role of non-traditional risk factors remains unclear and correction of anaemia or hyperparathyroidism has not been shown to improve outcomes in ESKD patients [80, 81, 82, 83, 84].

### 4.3 Chronic Inflammation

Chronic inflammation is an established CVD risk factor in the general population [85, 86, 87, 88, 89, 90]. Early epidemiological studies observed that acute phase reactants such as high sensitivity C-reactive protein (hsCRP) and interleukin (IL)-6 were strongly predictive of CVD outcomes. In 2010, the Emerging Risk Factors Collaboration confirmed the association between baseline hsCRP measurements with future CVD events and mortality with a magnitude of effect equivalent to that of LDL-C and blood pressure. ‘High-risk’ individuals with hsCRP levels >3 mg/L had an approximately 2-fold increased risk of adverse CVD outcomes. Statins were also found to have pleiotropic anti-inflammatory properties which appeared to extend their CVD benefits beyond cholesterol lowering [91]. The Use of statins in Primary prevention: an Intervention Trial Evaluating Rosuvastatin (JUPITER) study demonstrated that statin treatment reduced first-ever CVD events in healthy individuals with elevated hsCRP despite normal LDL-C levels. The Canakinumab Anti-Inflammatory Thrombosis Outcomes Study (CANTOS) trial demonstrated that human monoclonal antibody therapy against interleukin (IL)-1β reduced non-fatal MI, stroke and cardiovascular death in high-risk patients with elevated hsCRP (>2 mg/L) without any effects on blood pressure or lipid levels [92]. This was the first evidence that specifically targeting inflammation directly improves CVD outcomes. Currently research is focused on the nucleotide-binding leucine-rich repeat-containing pyrin receptor 3 (NLRP3) inflammasome which is directly activated by oxidised LDL/cholesterol particles and is the main driver for IL-1β/IL-6/CRP inflammatory pathway [93, 94].

**Fig. 8.**
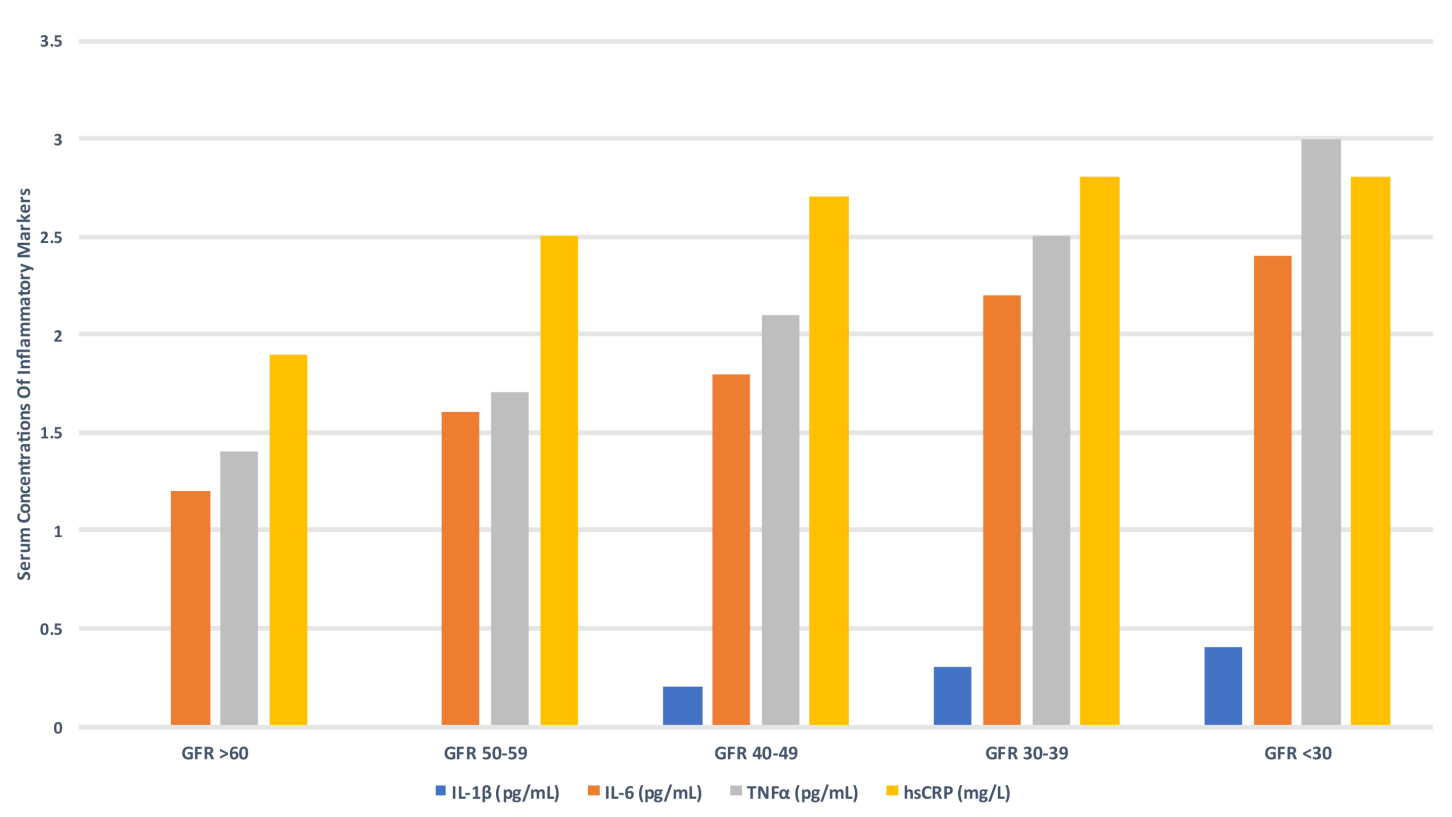
**Serum concentrations of inflammatory markers according to GFR (CRIC study) [99]**. GFR, glomerular filtration rate; IL-1β, interleukin-1β; TNFα, tumour necrosis factor-α; hsCRP, high sensitivity C-reactive protein.

Chronic inflammation in CKD is highly prevalent with increased hsCRP levels present in approximately 30–50% of adult and paediatric ESKD patients [95, 96, 97, 98]. Inflammation is inversely associated with kidney function and is part of overall immune dysregulation consisting of simultaneous activation and reduced function of selective components of innate and adaptive immunity (Fig. 8) [99, 100, 101, 102]. This results in chronic monocyte stimulation and increased production of pro-inflammatory cytokines and is further exacerbated by reduced renal clearance of pro-inflammatory mediators as kidney function declines. Monocytes form part of the initial innate immune response to entrapped LDL-C particles in the arterial wall and play key roles in the formation and progression of atherosclerotic plaques through the release of inflammatory pro-atherogenic cytokines such as interleukin (IL)-12 and interleukin (IL)-18 and recruitment of adaptive immunity (T- and B-lymphocytes) [87]. Monocytes are also implicated in the pathogenesis of myocardial fibrosis and subsequent development of heart failure with preserved ejection fraction [103].

Inflammation has a multifactorial aetiology in ESKD patients which includes: (1) uraemic toxins, (2) oxidative stress and cellular senescence, (3) hypoxia, fluid and volume overload, (4) gastrointestinal dysbiosis, (5) increased frequency of infections, (6) dialysis access (extracorporeal dialysis circuit, dialysis membranes and central venous catheters), (7) increased adipose tissue and adipokines. This may be further modified by genetic predisposition and epigenetic factors such as diet, lifestyle and environmental influences [104, 105, 106, 107].

**Fig. 9.**
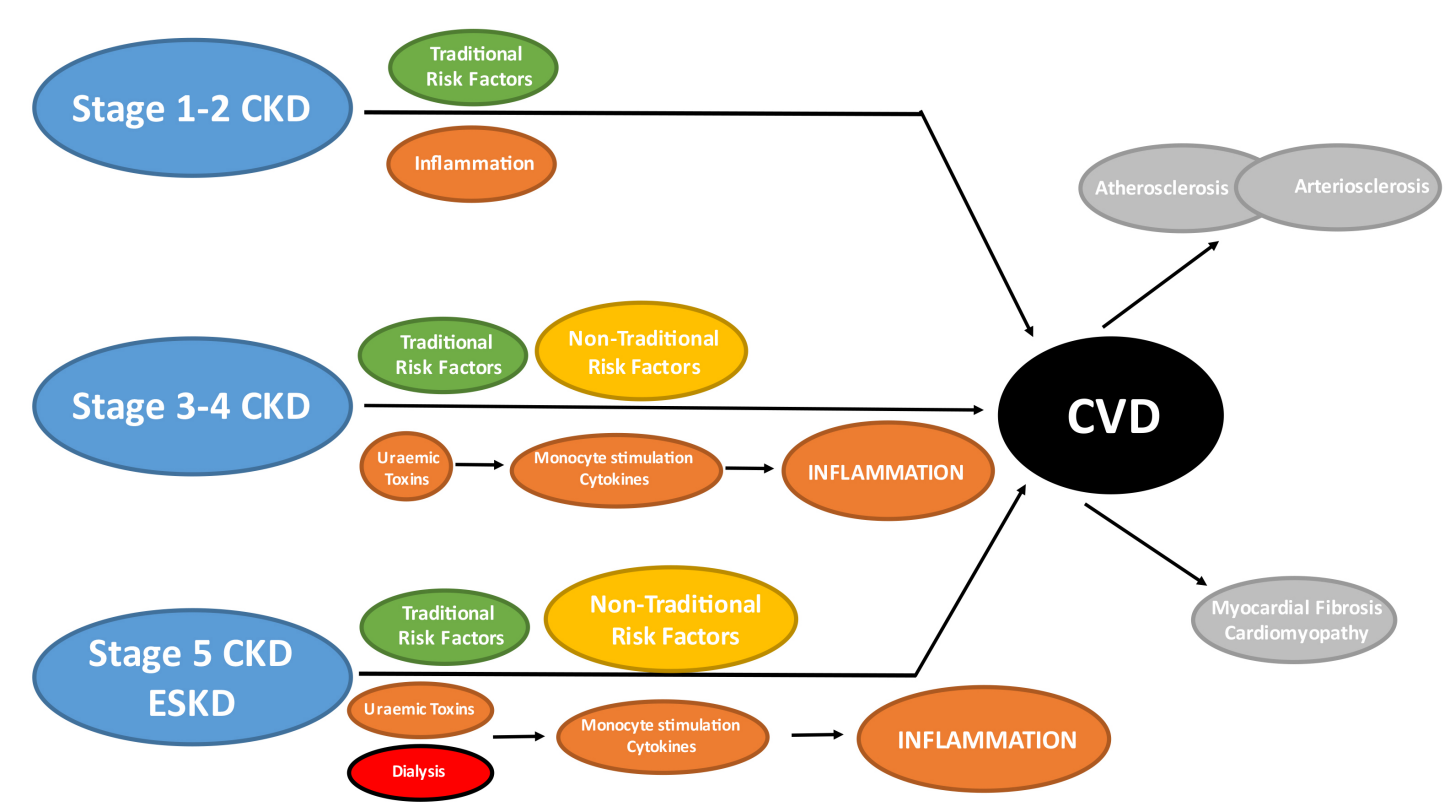
**Traditional & non-traditional risk factors in CKD**. Traditional risk factors are present at all stages of CKD but may have relatively less importance compared to non-traditional risk factors in more advanced CKD. Chronic inflammation is an established non-traditional risk factor in the general population which is not specific to CKD patients. However, stimulation of the innate immune system (monocytes) by uraemic toxins and dialysis itself results in chronic production of pro-inflammatory cytokines which contribute to the overall inflammatory burden and may have direct adverse effects upon the cardiovascular system. The cumulative effects of traditional, non-traditional risk factors and inflammation results in the development of arterial (atherosclerosis and arteriosclerosis) and cardiac (myocardial fibrosis) disease in CKD patients. CKD, chronic kidney disease; CVD, cardiovascular disease; ESKD, end-stage kidney disease.

Inflammation is a marker of poor prognosis in ESKD [108, 109, 110, 111, 112, 113, 114, 115, 116]. The International Dialysis Outcomes and Practice Patterns Study (DOPPS showed that hsCRP levels >3 mg/L in chronic dialysis patients were associated with a 1.6-fold increased risk of all-cause mortality [117]. The mechanism through which this occurs is unclear but is presumably due to increased CVD given the strong relationship between hsCRP and IL-6 with CVD outcomes and mortality in ESKD (Fig. 9).

Chronic inflammation presents an attractive therapeutic target for CVD risk reduction in ESKD [118, 119, 120]. A post-hoc analysis of CANTOS reported that the subset of patients with CKD (eGFR 30–60 mL/min/1.73 $m^{2}$) also achieved an 18% reduction in major cardiovascular events with canakinumab. Anakinra (IL-1 receptor antagonist) and rilonacept (IL-1 trap) improved endothelial function and inflammatory markers in chronic dialysis and stage 3–4 CKD patients. The recent phase II Reduction in Inflammation in Patients with Advanced Chronic Renal Disease Utilising Antibody Mediated IL-6 Inhibition (RESCUE) trial showed that IL-6 inhibition with ziltivekimab successfully reduced IL-6 and hsCRP levels in patients with stage 3–5 CKD (eGFR 10–60 mL/min/1.73 $m^{2}$) [121]. A large-scale randomised trial (ZEUS; ClinicalTrials.gov NCT05021835) assessing the effects of ziltivekimab upon CVD outcomes in CKD patients is currently underway.

### 4.4 Dialysis Modality

Haemodialysis (HD) and peritoneal dialysis (PD) are the two main dialysis modalities available worldwide. The question of whether CVD risk is modified by dialysis modality remains a subject of controversy. The intermittent nature of HD leads to rapid volume shifts, myocardial stunning, rapidly altered drug concentrations, dyskalaemias and other electrolyte disorders which exacerbate underlying CAD and increase risk of arrhythmias and sudden death [122, 123]. Exposure to the extracorporeal dialysis circuit is also associated with more rapid loss of residual renal function [124]. The arteriovenous fistula (AVF) used for HD access also results in a hyperdynamic circulation which predisposes to development of cardiac failure and can exacerbate atherosclerosis by promoting turbulent arterial blood flow [125]. The continuous nature of PD is associated with superior preservation of residual renal function and may minimise CVD risk by avoidance of electrolyte peaks and troughs and providing better haemodynamic stability [126]. However, PD patients are exposed to significant dialysate glucose loads which encourages insulin resistance, proatherogenic lipid profile, metabolic syndrome and deposition of advanced glycation end-products (AGEs) [127].

Previous attempts at randomised controlled trials to answer this important question have been complicated by lack of statistical power and poor recruitment because patient and physician preferences play crucial roles in choice of dialysis modality [128, 129]. A randomised trial comparing HD and PD has been ongoing in China for quite some time (ClinicalTrials.gov NCT01413074) but the results of this are not yet available [130]. Retrospective observational studies comparing all-cause mortality between HD and PD are conflicted (Table 5, Ref. [131, 132, 133, 134, 135, 136, 137, 138, 139, 140, 141, 142, 143, 144, 145, 146, 147, 148]). Some have shown a superior time-dependent benefit of PD usually only for the first 2 years of treatment and restricted to younger patients without co-morbidities [131, 132, 133, 134, 135, 136, 147, 148]. Others have found either no difference between HD and PD while others suggest that HD confers superior survival particularly in older patients with cardiac failure or diabetes [137, 138, 139, 140, 141, 142, 143, 144, 145, 146].

**Table 5.** **Retrospective observational studies comparing mortality outcomes between dialysis modality (HD vs PD) [131, 132, 133, 134, 135, 136, 137, 138, 139, 140, 141, 142, 143, 144, 145, 146, 147, 148]**.

Favours PD*	No difference	Favours HD
Collins 1999 [132]	Mehrota 2011 [139]	Bloembergen 1995 [140]
Winkelmeyer 2002 [131]	Wong 2018 [141]	Foley 1998 [145]
Heaf 2002 [137]		Ganesh 2003 [143]
Vonesh 2004 [135]		Stack 2003 [142]
McDonald 2009 [133]		Jaar 2005 [146]
Weinhandl 2010 [136]		Sens 2011 [144]
Lukowsky 2013 [134]		Kim 2014 [147]
Kumar 2014 [138]		Thiery 2018 [148]

PD, peritoneal dialysis; HD, haemodialysis. *Studies favouring PD generally found superior survival only during the initial 1–2 years of dialysis therapy and mainly restricted to specific sub-groups (younger patients without medical comorbidities).

Very few studies have specifically compared CVD outcomes between HD and PD patients. The ANZDATA registry reported that incident PD patients had significantly higher CVD mortality compared with HD after the first year of treatment [149]. This increased risk of CVD death was predominantly driven by MI. Similar results were reported in a Korean ESKD cohort which observed increased risk of major adverse cardiovascular events (MACE) in patients starting PD beyond the first year of treatment [150]. In contrast to these, a Taiwanese-based cohort study found that incident HD patients were at greater risk of developing *de novo* CAD compared to PD [151]. A sub-study of the Spanish prospective NEFRONA cohort reported that dialysis modality had no impact upon CVD events, mortality or carotid artery atherosclerosis in a matched population of HD and PD patients [152]. A meta-analysis of 5 cohort studies (n = 47,062) found a similar incidence of MACE between patients initiated on either HD or PD [153].

Inherent weaknesses such as ascertainment bias, selection bias and immortal time bias may explain the conflicting results of these retrospective studies [154, 155, 156]. In addition, there is often significant variation in clinical practice patterns and guidelines regarding dialysis modality [157]. PD patient populations are also heterogenous and may include younger healthier patients more likely to undergo renal transplantation as well as older patients with limited life expectancy (indication bias). Further confounding can be introduced by patients switching dialysis modality (usually PD to HD) [158]. Therefore, the comparative effects of HD and PD upon CVD and survival remains unclear.

## 5. Treatment of CAD Risk Factors in ESKD

Medical therapy is highly effective at reducing cardiovascular events and mortality in stable coronary artery disease in the non-ESKD population. Establishing the efficacy of medical therapy in the ESKD population is challenging because, as a group, the ESKD population has been severely under-represented in trials, and the relative contribution of atherosclerosis to CVD in the ESKD is small.

### 5.1 Hypertension: Blood Pressure Targets & Anti-Hypertensive Agents

Hypertension is one of the most powerful CVD risk factors and is a leading contributor to CVD mortality and morbidity worldwide [159, 160, 161]. The definition of hypertension has evolved over time with lower blood pressure (BP) targets now being suggested in both the general and pre-dialysis CKD populations based largely upon the Systolic Blood Pressure Intervention Trial (SPRINT) showing that intensive BP lowering (SBP <120 mmHg) significantly decreased MACE and all-cause mortality [162, 163, 164, 165, 166].

The 2021 KDIGO guidelines currently recommend target BP <120/80 mmHg for all non-dialysis CKD patients if tolerated [163, 166]. The applicability of current guidelines in ESKD patients remain questionable. The SPRINT trial included a significant number of CKD patients (n = 2646; eGFR <60 mL/min/2.73 $m^{2}$) but only sixteen of these were on dialysis. Randomised trials to inform BP targets or support BP lowering are lacking in ESKD and large observational studies have reported that both lower and higher BP values are associated with poor outcomes in dialysis patients [76]. This “J” or “U” shaped relationship between BP and mortality in ESKD patients describes increased mortality with either SBP <110 mmHg or >170 mmHg [167]. Recommendations from the European Renal Association-European Dialysis and Transplant Association (ERA-EDTA) and National Kidney Foundation Kidney Disease Outcomes Quality Initiative (KDOQI) have reflected these concerns by abstaining from providing a preferred BP target in dialysis patients [168, 169].

The Blood Pressure in Dialysis (BID) study compared intensive BP (pre-dialysis SBP target of 115–140 mmHg) to standard control (pre-dialysis SBP 155–165 mmHg) in ESKD patients [170]. This pilot study recruited 126 chronic HD patients and showed no difference in CVD endpoints between groups. In addition, intensive BP lowering was associated with increased risk of AVF thrombosis, intra-dialytic hypotension, hospitalisation, nausea/vomiting and cramps which highlights the unique harms of blood pressure lowering in dialysis patients.

A meta-analysis of 1679 ESKD patients from 8 randomised controlled trials reported that BP treatment (with any agent) was associated with a 29% and 20% reduction in CVD and all-cause mortality respectively. However, the mean BP reductions achieved were small (4.5 mmHg systolic and 2.3 mmHg diastolic) and no optimal BP targets were identified [171]. A subsequent meta-analysis of 1202 ESKD patients from 5 randomised trials also reported that BP treatment reduced MACE by 30% [172]. Neither meta-analysis was able to determine if these observations were driven by actual BP lowering or specific drug class effects (such as the negatively chronotropic and inotropic effects of β-blockers).

Further important considerations in ESKD are that BP measurements often fluctuate and depend on whether they were recorded pre-dialysis, post-dialysis or during the inter-dialytic period. The significance of timing of BP measures in dialysis patients remains unclear and there is also often poor correlation between intra- and inter-dialytic BP readings [173]. Efforts to characterise inter-dialytic BP profile have shown that BP values progressively rise leading up to the next dialysis session [174]. Results from observational studies are conflicting with some studies reporting increased mortality risk with low pre- and post-dialysis BP readings while others have found no relationship at all [175, 176]. Other BP measurements including post-dialysis pulse pressure, intra- or inter-dialytic BP variability and inter-dialytic (over intra-dialytic) BP readings have been put forward as predictors of CVD outcomes or mortality but these are inconsistent [175, 176, 177, 178].

In summary, there is only weak evidence to support BP lowering and no evidence to support a specific BP target in ESKD patients. There is no clear choice for optimal class of anti-hypertensive agents in ESKD.

### 5.2 Hypertension: RAAS Blockade

Limited data suggests that RAAS blockade with angiotensin-converting enzyme inhibitors (ACEi)/ARBs may be favourable in chronic dialysis patients but are often limited by hyperkalaemia [179]. Renin-angiotensin-aldosterone system (RAAS) blockade with ACEi or angiotensin II receptor blockers (ARB) are the cornerstone of hypertension management in non-dialysis CKD due to their benefits upon renal outcomes [180, 181, 182, 183]. RAAS blockade may also have favourable effects upon the kynurenine pathway (KP) which is the major catabolic pathway for tryptophan degradation. Altered KP metabolism in CKD due to chronic inflammation results in increased synthesis and accumulation of biologically active tryptophan metabolites which in turn have numerous harmful effects on the body [184, 185, 186]. Serum levels of KP metabolites are predictive of subclinical atherosclerotic disease and CVD events in patients with advanced CKD [187]. Observational studies have suggested that serum kynurenine levels in CKD patients may be attenuated by RAAS blockade [188].

The CVD benefits of RAAS blockade in CKD populations have yet to be firmly established and high-quality evidence in ESKD remains lacking [189, 190, 191, 192]. Neither the Fosinopril in Dialysis (FOSIDIAL) nor the Olmesartan Clinical Trial in Okinawa Patients Under Okinawa Dialysis (OCTOPUS) studies demonstrated any CVD benefits with fosinopril or olmesartan in chronic HD patients respectively [193, 194]. On the other hand, two randomised trials of ARBs in Japanese HD patients and the addition of telmisartan to ACEi in HD patients with cardiac failure showed significant reductions in CVD events, mortality and cardiac-failure related hospitalisations [195, 196, 197]. There are currently no randomised trial data available in PD although a retrospective analysis of the USRDS suggests that ACEi/ARB use is associated with decreased risk of fatal CVD outcomes in PD patients [198].

### 5.3 Hypertension: Potential Role of β-Blockers

β-blockers are an established therapy for cardiac failure in the general population [199]. Post-hoc analyses of the Cardiac Insufficiency Bisoprolol Study II (CIBIS-II) and the Metoprolol CR/XL Randomised Intervention Trial in chronic HF (MERIT-HF) suggest that cardiac failure patients with CKD may also derive similar CVD benefits from β-blocker therapy [200, 201]. Large randomised trials on β-blocker therapy in ESKD are lacking [202]. Attempted trials on β-blockers in chronic dialysis patients with normal cardiac function such as the Hypertension in Hemodialysis Patients Treated With Atenolol or Lisinopril (HDPAL) and β-Blocker to Lower Cardiovascular Dialysis Events (BLOCADE) were terminated early due to safety concerns and poor recruitment [203, 204].

It should be noted that in contrast to CKD patients with normal cardiac function, β-blockers may improve CVD outcomes in symptomatic ESKD patients with established cardiac failure [205, 206]. Carvedilol improved left ventricular function and was associated with fewer deaths and hospital admissions compared to placebo in chronic dialysis patients (n = 114) with dilated cardiomyopathy and New York Heart Association (NYHA) symptom scores >1, albeit in a relatively small randomised trial.

There are no placebo-controlled trials available for β-blocker therapy in asymptomatic dialysis patients. The HDPAL trial compared atenolol with lisinopril in chronic HD patients with hypertension and established LVH [203]. HDPAL reported that atenolol was associated with decreased CVD morbidity and hospitalisations but was terminated prematurely due to safety concerns. Observational data have suggested that β-blockers may reduce mortality and cardiac events in ESKD patients [176, 207, 208]. A retrospective analysis of the USRDS reported that β-blockers were associated with the lowest mortality of all anti-hypertensive classes in chronic dialysis patients. Other studies (including DOPPS) have identified reduced sudden cardiac death with β-blocker therapy even after adjustment for comorbidity burden and dialysis prescription.

Evidence suggests that β-blocker dialysability may play an important role in treatment efficacy [209, 210]. Less dialysable β-blockers such as bisoprolol, carvedilol and propranolol have been observed to have mortality benefits compared to highly dialysable agents such as metoprolol and atenolol. A Taiwanese cohort study of over >20,000 chronic dialysis patients reported that bisoprolol was superior to carvedilol in reducing MACE and all-cause mortality over a 2-year period. Possible mechanisms for the superiority of less dialysable β-blockers include BP lowering, anti-arrhythmogenic properties and blunted sympathetic activity which may minimise intra-dialytic haemodynamic instability.

### 5.4 Lipid-Lowering Therapy

CKD is associated with complex qualitative and quantitative alterations to lipid metabolism and uraemic dyslipidaemia is classically described as raised triglycerides (TG), low high-density lipoprotein cholesterol (HDL-C) and normal total cholesterol concentrations [211]. The pro-atherogenic profile of ESKD is also further modified by uraemia and other factors such as chronic inflammation which alters the concentrations and compositions of lipoprotein particles [16, 212, 213, 214, 215]. HDL-C appears particularly susceptible to inflammatory modifications and loses its anti-atherogenic functions while gaining pro-inflammatory and pro-atherogenic properties. Modified acute phase HDL-C is an independent predictor of adverse clinical outcomes in ESKD patients. In addition, urea-related modification (also known as carbamylation) may also increase the pro-atherogenic properties of LDL-C [216].

Low-density lipoprotein cholesterol (LDL-C) reduction with β-Hydroxy β-methylglutaryl-CoA (HMG-CoA) reductase inhibitors (statins) is well-established for CVD risk reduction in the general population [217, 218]. However, statin therapy remains controversial in ESKD. Three major randomised controlled trials have assessed statin therapy in dialysis patients [34, 219, 220]. The 4D and A Study to Evaluate the Use of Rosuvastatin in Subjects on Regular Haemodialysis (AURORA) trials both failed to show that statins reduced CVD events or mortality in chronic HD patients despite an impressive LDL-C lowering effect (approximately 40%). The Study of Heart and Renal Protection (SHARP) trial was the largest randomised statin trial in CKD (n = 9270) of which roughly one-third were chronic dialysis patients. SHARP reported a 17% reduction in major atherosclerotic events but this benefit did not extend to the dialysis sub-cohort and overall mortality was unchanged. Contrary to these randomised trial data, a large propensity score-matched analysis (n = 65,404) from a Korean health insurance registry reported that statin therapy was associated with reduced all-cause mortality especially when used in combination with ezetimibe [221]. Several large meta-analyses have concluded that statins reduce major CVD events and mortality in pre-dialysis CKD patients but with progressively smaller relative risk reductions as eGFR declines and little benefit of evidence in ESKD [222, 223].

Concerns have been raised that statins may accelerate the vascular calcification process in ESKD but the effects and location of these appear atypical of calcifications associated with classical CAD [224, 225]. Increased chronic inflammation in ESKD patients may also lead to ‘statin resistance’ by activating intracellular cholesterol synthesis which is only partially attenuated by conventional statin dosing and leads to intracellular lipid accumulation within the arterial wall despite reduction in serum LDL-C levels [226].

Current randomised trial data does not support statin therapy for improving CVD outcomes in ESKD patients despite LDL-C reduction with these agents. The 2013 Kidney Disease: Improving Global Outcomes (KDIGO) practice guidelines recommend statins for CVD risk reduction in pre-dialysis CKD patients but propose that statins should not be initiated in ESKD patients receiving chronic dialysis therapy [227]. Further study is required to determine if therapies targeting low HDL-C and high TG as well as lipoprotein(a) abnormalities may provide CVD benefits in ESKD patients. Novel agents such as the proprotein convertase subtilisin/kexin type 9 (PCSK9) inhibitors or cholesteryl ester transfer protein inhibitors (CETP) may be of interest in this regard [228, 229].

In summary, medical therapies for traditional Framingham risk factors are attenuated in ESKD patients. This is explained in part by the relatively low contribution of CAD to CVD in comparison to other pathologies such as myocardial fibrosis and vascular calcifications in advanced CKD and ESKD. The evidence base is also limited because CKD and ESKD patients are under-represented in large clinical trials leading to extrapolation of data from the general population [202]. The presence of non-traditional risk factors may further amplify CVD risk in ESKD.

## 6. Coronary Artery Disease Screening and Diagnosis

As with the non-ESKD population there is no strong evidence suggesting that screening asymptomatic CAD improves outcomes in ESKD. Screening is particularly relevant and best studied in stable ESKD patients wait-listed for transplantation in whom “missed” CAD may lead to suboptimal patient and graft outcomes [230].

There is considerable variation between guidelines and institutions on the optimal modality and timing of screening for stable CAD in ESKD patients being assessed for kidney transplant, reflecting the lack of robust randomised data to guide clinical practice. Findings from non-ESKD cohorts have often been extrapolated to ESKD patients. Non-invasive screening tests also underperform in ESKD compared to the general population [231].

## 7. Non-Invasive Testing

Non-invasive screening options for stable CAD include functional tests such as exercise stress testing (EST) dobutamine stress echocardiography (DSE), myocardial perfusion scan (MPS) and anatomical tests including coronary artery calcification scoring (CAC) and computerised tomography (CT) coronary angiography (CTCA).

### 7.1 Functional Tests

EST performs poorly in ESKD patients [232, 233, 234]. Approximately 50–90% of dialysis patients are unable to achieve target heart rate with exercise and inability to complete exercise testing is a poor prognostic sign.

MPS and DSE are the most accurate non-invasive screening modalities for asymptomatic CAD in ESKD patients [231]. Abnormal results in both investigations predict revascularisation and MACE in dialysis patients [235, 236, 237]. However, the sensitivity and specificity of both these modalities are modest at best. Local expertise and availability also play an important role in choice of screening modality.

### 7.2 Myocardial Perfusion Scanning

MPS is the most utilised screening tool among potential transplant candidates in the USA and UK [238, 239]. MPS uses a radionucleotide tracer to map cardiac blood flow and detect areas of hypoperfusion. Images are collected before, during, and after the addition of a coronary vasodilator, commonly dipyridamole or adenosine, providing functional information. In a large Cochrane analysis, the pooled sensitivity and specificity for MPS in predicting coronary lesions of >70% stenosis was 67% and 77% respectively [231]. However, the usefulness of MPS is highly dependent on the number of coronary vessels that are diseased. In a prospective trial of 482 kidney transplant candidates who underwent both MPS and coronary angiography, MPS was unhelpful in identifying patients with single vessel disease [240].

Single-Photon Emission Computerized Tomography (SPECT) is less commonly used to screen for CAD in ESKD because of high false positive rates and poor correlation between SPECT results and angiography findings [241, 242, 243]. This mismatch between abnormal SPECT results and angiographic findings limits the utility of SPECT as a CAD screening tool in ESKD patients. It has been hypothesised that abnormal SPECT results may represent true subendocardial ischaemia without coronary artery stenosis which in turn correlates with MACE outcomes [243, 244].

### 7.3 Dobutamine Stress Echocardiography

DSE has enjoyed increasing popularity since 2010 for CAD screening in potential transplant candidates [238]. DSE has moderate sensitivity (76%) and specificity (88%) for detecting coronary artery stenoses of >70% in dialysis patients [231]. DSE and MPS share greater than 85% concordance for the detection of reversible and fixed defects. The post-test probability of CAD after a negative result is lower with DSE than MPS which suggests that DSE may be a more useful initial test when both are available.

A major limitation of DSE is the high prevalence of LVH (75%) and left ventricular systolic dysfunction (28%) in ESKD patients [245, 246, 247]. These pre-existing cardiac abnormalities, fluctuations in volume status and other factors such as age and diabetes are associated with higher incidence of abnormal test results and subsequent loss of specificity [245]. Local expertise is also particularly important as DSE is heavily operator dependent.

## 8. Anatomical Tests

### Coronary Artery Calcium Scoring and CT Coronary Angiography

CACs has been recommended by the European Society of Cardiology and American College of Cardiology as a “rule out” test for patients with an intermediate risk of CAD [248]. The reported sensitivities and specificities of CAC in ESKD patients appear similar to that of MPS and DSE [249]. A CAC score of >400 has a reported sensitivity and specificity of 67% and 77% respectively for detecting coronary lesions >50% in ESKD patient. CAC scores of >500 are also independent predictors of CVD events and mortality in chronic dialysis patients [250, 251]. CACs has the advantage of not requiring contrast or a nuclear medicine facility. However, the high prevalence of elevated coronary calcification in chronic dialysis patients (approximately >80%) may limit the utility of this test in ESKD [252].

CTCA is now recommended as a first-line test for the investigation of CAD by the European Society of Cardiology, American College of Cardiology/American Heart Association, and the National Institute for Health and Care Excellence. Indraratna *et al*. [253] demonstrated that the initiation of statin therapy for non-obstructive coronary disease detected on CTCA reduced major cardiovascular events over a 5-year period. While there are concerns around the utility of predicting obstructive disease in ESKD due to a high burden of coronary artery calcification, a study by Mao *et al*. [254] in pre-transplant patients showed that 36% of patients had normal coronary artery arteries with a calcium score of 0. In this study, obstructive coronary artery disease was excluded in 70% of patients, albeit in a small sample group. There is the potential of CTCA to evolve into a first-line screening test for ESKD patient, although those with a high coronary calcium score or presence of obstructive disease would likely need additional functional testing.

## 9. Invasive Testing

### 9.1 Coronary Angiography

Invasive coronary angiography remains the gold standard for the diagnosis of coronary artery disease [255]. Refined intravascular imaging techniques including intravascular ultrasound (IVUS) and optical coherence tomography (OCT) have provided valuable information about plaque composition and calcium burden over and above conventional coronary angiography. Coronary angiography can be performed with ultra-low volumes of contrast by using only angiographic views, confirming catheter engagement with intracoronary saline injections, and then using either IVUS or invasive functional testing to determine the haemodynamic significance of any lesions [256]. Invasive functional assessments using fractional flow reserve (FFR) and instantaneous wave free ratios (iFRs) have become standard of care regarding treatment decisions for coronary artery disease but are largely unvalidated in the ESKD population.

### 9.2 Coronary Physiology in Dialysis Patients

Pressure wire measurements including FFR and iFR assess the pressure difference across a coronary artery lesion during invasive coronary angiography. They are expressed as a ratio between the distal coronary artery pressure divided by proximal aortic pressure with a haemodynamically significant result defined as FFR ≤0.80 and iFR ≤0.89 [257]. FFR is obtained under maximal hyperaemia with adenosine whereas iFR is obtained at rest. They are the gold standard to assess the haemodynamic significance of coronary lesions in both European and American guidelines [255, 258]. However, their role in ESKD patients remains controversial as there is very limited evidence. Major landmark studies supporting FFR or iFR include Deferral of Percutaneous Coronary Intervention (DEFER) trial, Fractional Flow Reserve versus Angiography for Multivessel Evaluation (FAME) study, FAME 2 study, The Instantaneous Wave-free Ratio versus Fractional Flow Reserve in Patients with Stable Angina Pectoris or Acute Coronary Syndrome (iFR-SWEDEHEART) and Functional Lesion Assessment of Intermediate Stenosis to Guide Revascularisation (DEFINE-FLAIR) trials [257, 259, 260, 261, 262]. Of these, only FAME 2 reported ‘renal insufficiency’ and the generalisability of the trial results to ESKD patients remains limited due to only 1.8% of patients in the percutaneous coronary intervention arm having renal insufficiency [262].

FFR and iFR measurements are affected by factors including microsvascular dysfunction, reduced arterial compliance, left ventricular impairment and complex (diffuse and calcific) CAD [263, 264, 265, 266, 267, 268]. CKD and ESKD patients have higher rates of microvascular disease, more complex CAD and concomitant cardiac disease which affects physiological results [269, 270]. Indeed, worsening kidney function is associated with higher FFR and iFR values [271, 272].

The performance of FFR is suboptimal in dialysis patients. It is known that FFR correlates well with anatomical measurements including quantitative coronary angiography (QCA) in the general population [273, 274]. However, lesion severity quantified by FFR has poor correlation with minimal lumen diameter or percentage stenosis on QCA in HD patients [275]. Nevertheless, in a separate study examining FFR results against ischaemia detected on MPS, the optimal FFR cut-off for ischaemic significance was similar between HD patients and other CAD patients [276]. Patients on dialysis also have higher rates of discordance between FFR and iFR. Multiple studies have demonstrated that resting physiological measurements show lower results than FFR in HD patients [277, 278].

Evidence strongly supports pressure wire-guided revascularisation as it improves clinical outcomes, however again the evidence in dialysis populations is poor. The DEFER trial established that it was safe to defer revascularisation of FFR-negative coronary lesions [261]. A similar study in HD patients observed significantly higher rates of MACE and target vessel failure (TVF) following deferral of revascularisation (FFR >0.80) [279]. In a separate study comparing outcomes between FFR-guided revascularisation (FFR ≤0.80) and non-intervention, functional ischaemia was associated with a poorer prognosis despite revascularisation in pre-dialysis CKD and ESKD patients [280]

Overall, the performance of invasive pressure wire measurements in ESKD patients remains controversial. A combined FFR-iFR approach is beneficial to maximise accuracy and the physiological result should be corroborated with anatomical measurements.

## 10. Coronary Revascularisation

### 10.1 Coronary Revascularisation and Stable CAD

There is no evidence that revascularisation of stable CAD improves clinical outcomes in patients with or without CKD [281, 282, 283, 284, 285, 286]. Several landmark trials including Coronary Artery Revascularisation Prophylaxis (CARP), Clinical Outcomes Utilising Revascularisation and Aggressive Drug Evaluation (COURAGE), Dutch Echocardiographic Cardiac Risk Evaluation Applying Stress Echo-V (DECREASE-V), Bypass Angioplasty revascularisation Investigation 2 Diabetes (BARI 2D), Detection of Ischaemia in Asymptomatic Diabetics (DIAD) and the International Study of Comparative Health Effectiveness with Medical and Invasive Approaches (ISCHEMIA) have demonstrated that revascularisation was not superior to optimal medical therapy for stable CAD in several high-risk populations. An important observation is that coronary physiology was not used to guide revascularisation in these trials. FAME, FAME 2 and DEFER trials established that coronary physiology was superior to angiography alone and is the gold standard for informing revascularisation decisions [261, 262].

The ISCHEMIA-CKD trial was a sub-study of the ISCHEMIA trial and included 777 patients with eGFR <30 mL/min/1.73 $m^{2}$ and evidence of moderate-severe ischaemia on non-invasive testing [287]. Of these, 194 patients were chronic dialysis patients on the transplant wait-list. Study participants were randomised to either invasive angiography and revascularisation (if deemed appropriate) or optimal medical therapy alone. ISCHEMIA-CKD reported no difference in the composite outcome of mortality or non-fatal MI after a median 2.2 year follow-up period regardless of transplant wait-list status [288]. Patients randomised to the angiography arm had an almost 4-fold increased risk of stroke and accelerated progression to dialysis. Criticisms of the ischemia-ckd trial were that only 80% of patients had obstructive coronary disease that was confirmed on invasive angiography, and there was significant proportion of revascularisation in the primary medical therapy arm. However, a recent meta-analysis of 8 trials (including ISCHEMIA-CKD) concluded that revascularisation did not improve all-cause or CVD mortality in ESKD patients [289]. As such a strong argument for upfront medical therapy rather than a primary invasive approach for the assessment and treatment of CAD in the ESKD population is favoured.

### 10.2 Coronary Revascularisation and ACS

Primary PCI is indicated in patients with ESKD and STEMI. However, the role for an early invasive strategy for NSTEACs is less clearly defined. Most data supporting an early invasive role for the treatment of non-STEACS is observational, as there is an absence of randomised data in the ESKD population. Registry data from the United Kingdom strongly supported and early invasive strategy for the management of NSTEACS, including those with an eGFR of <30 mL/min/1.73 $m^{2}$ [290]. Only a small portion of this group had an eGFR of <15 mL/min/1.73 $m^{2}$, and there was no separate analysis performed on this subgroup. A meta-analysis of 10 separate studies demonstrated heterogeneity within the benefit of an early invasive strategy versus medical therapy, with an overall combined benefit in those with an eGFR <30 mL/min/1.73 $m^{2}$ [291]. The number of ESKD patients included again was small, and not separately analysed.

### 10.3 Percutaneous Coronary Intervention versus Surgical Revascularisation

CKD is associated with poor outcomes following coronary artery bypass grafting (CABG) and PCI, even in contemporary trials. The Evaluation of XIENCE Versus Coronary Artery Bypass Surgery for Effectiveness of Left Main Revascularisation (EXCEL) trial compared outcomes from contemporary PCI with 3rd generation drug-eluting stent technology against contemporary CABG for left main coronary artery disease with low to intermediate anatomical complexity [292]. Those with pre-existing CKD, defined as an eGFR <60 mL/min/1.73 $m^{2}$ had a combined event rate of death, myocardial infarction or stroke of 20.8% compared with 13.5% with no CKD. There was no difference in the event rate between CABG and PCI [292].

Kumada *et al*. [293] presented 10-year follow-up data on 997 HD patients who underwent either CABG (210 patients) or PCI (787 patients). This was a non-randomised study using a combination of bare-metal and early generation drug-eluting stent technology. The hazard ratio for MACE was 0.69 (95% confidence interval 0.54–0.87) favouring CABG.

The Future Revascularisation Evaluation in Patients with Diabetes Mellitus: Optimal Management of Multivessel Disease (FREEDOM) trial demonstrated a significant benefit of CABG over PCI in diabetic population, with a 27% relative risk reduction in MACE at a median follow-up of 3.8 years [294]. Thus, PCI has made up ground on CABG with later generation drug-eluting stents, but there still may be an advantage of CABG over PCI with the diabetic population and those with high degrees of anatomical complexity.

## 11. Pre-Transplant CAD Screening

Perhaps the greatest controversy in the ESKD population remains around pre-transplant screening. Current recommendations for screening of stable CAD in ESKD patients and prior to kidney transplantation are consensus based with the goal being to guide the need for invasive coronary angiography (Table 6, Ref. [230, 240, 295, 296, 297, 298, 299]). The level of evidence is generally poor and there is clear variability between guidelines. Most clinical recommendations are informed by observational studies with grade C level evidence. There is also no evidence to support one screening modality over the other. Therefore, appropriate risk stratification, local expertise and resource availability become important factors for stable CAD screening in ESKD patients.

**Table 6.** **Summary of transplant guidelines recommendations on CAD screening**.

	Year	Population	Recommendation
K/DOQI [230]	2005	New to dialysis	All patients require assessment for CVD and screen for traditional and non-traditional RFs at the initiation of dialysis.
			Baseline echocardiogram within 3 months of starting dialysis once dry weight is achieved, and 3 yearly thereafter.
	2005	Transplant waitlist (asymptomatic)	Screening interval depends on risk:
			Diabetes or known CAD (and not revascularized) - Every 12 months.
			Non-diabetic but “high risk” (>20% 10 yr CV event risk by the Framingham data, known CAD, PVD or a LVEF <40%) - Every 24 months.
			Not high risk - Every 36 months.
Canadian Society of Transplantation consensus guidelines [295]	2005	Transplant candidates (asymptomatic)	All patients should be assessed for IHD with a minimum history, exam, ECG and CXR (Grade A).
			Non-invasive testing for asymptomatic patients with known CAD, diabetes or who have 3 or more of the listed risk factors (Grade B).
			- Age >50 years, prolonged CKD, family history CAD, Smoking history, dyslipidaemia, HTN.
			All patients with a positive test should be referred for angiography (Grade B).
			Very high-risk patient should be referred for angiography even with a negative stress test (Grade C).
			Re-evaluation should occur annually in high-risk patients (Grade C) which includes history, exam, ECG and non-invasive testing (Grade C).
Renal Association Clinical Practice guideline on the Assessment of Potential Kidney Transplant Recipients [296]	2011	Transplant candidates (asymptomatic)	Until better evidence emerges, screening tests may be best used to identify high-risk patients for exclusion from the transplant waiting list (2C).
			No modality specified.
American Heart Association and American College of Cardiology (AHA/ACC) Scientific Statement [240]	2012	Transplant candidates (asymptomatic)	Consider non-invasive testing in the presence of 3 or more risk factors;
			- Diabetes mellitus, prior CVD, >1 year on dialysis, LVH, age >60 years, smoking, hypertension, and dyslipidemia. (Class IIb; Level of Evidence C).
			- Testing modality not specified but MPS and DSE discussed.
			Utility of periodic screening on the waiting list is “uncertain” (Class IIb; Level of Evidence C).
KHA-CARI Recipient assessment for transplantation [297]	2013	Transplant candidates (asymptomatic)	All transplant candidates should be screened for cardiovascular risk factors (1B).
			Candidates with a low clinical risk do not require stress testing (2B).
			Candidates with moderate or high risk of CAD should have stress testing prior to transplantation (2B).
			- Modalities referenced include; Dipyridamole-thallium testing or stress echocardiogram, preferably without concurrent B-blocker therapy (1B).
			Repeat testing is suggested without specified intervals (2C).
			Coronary angiography should be considered for anyone with an abnormal screening test (1B).
European Renal Best Practice Guidelines [298]	2015	Transplant candidates (asymptomatic)	Basic physical examination, resting ECG and chest-X ray are a sufficient standard work-up in asymptomatic low-risk kidney patients (1C).
			Suggest standard exercise tolerance test and cardiac ultrasound be performed in high-risk patients (older age, diabetes, history of cardiovascular disease).
			In patients with a true negative test, further cardiac screening is not indicated (1C).
			Non-invasive stress imaging with MPS or DSE should be undertaken for high-risk patients with a positive or inconclusive exercise tolerance test (1C).
			All patients with a positive test should be referred for angiography.
KDIGO clinical practice guideline [299]	2020	Transplant candidates (asymptomatic)	Evaluate all candidates for cardiac disease with history, physical examination, and ECG (Not graded).
			Non-invasive testing is recommended based on risk factors or poor functional capacity (Grade 2C) – no specific modality preferred.
			Asymptomatic candidates with known CAD should *not* be revascularized exclusively to reduce perioperative cardiac events (1B).
			Asymptomatic candidates who have been on dialysis for at least two years should undergo echocardiography (2D).

CAD, coronary artery disease; KDOQI, Kidney Disease Outcomes Quality Initiative; CVD, cardiovascular disease; CV, cardiovascular; PVD, peripheral vascular disease; LVEF, left ventricular ejection fraction; IHD, ischaemic heart disease; ECG, electrocardiogram; CXR, chest xray; CKD, chronic kidney disease; HTN, hypertension; LVH, left ventricular hypertrophy; KHA, Kidney Health Australia; CARI, Caring for Australians with Renal Impairment; MPS, myocardial perfusion scan; DSE, dobutamine strss echocardiography; KDIGO, Kidney Disease Improving Global Outcomes.

The benefits of revascularisation therapy for stable CAD in ESKD patients was first demonstrated in a randomised trial of type I diabetic ESKD patients being considered for kidney transplant [300]. However, this study was limited by small sample size (n = 26) and conducted during an era in which optimal medical management consisted of only calcium channel blocker and aspirin.

The Access to Transplant and Transplant Outcome Measures (ATTOM) study reported that non-invasive screening did not improve MACE outcomes or reduce mortality in kidney transplant candidates (n = 1053) [239]. This retrospective analysis also observed that abnormal screening results resulted in significant delays to transplant wait-listing by approximately 4 months with none of these patients actually requiring revascularisation.

ESKD patients being worked up for transplantation are a unique sub-group with additional considerations such as peri-operative risk, avoidance of unnecessary waiting time, economic utility and resource allocation. Screening is probably justified in these patients but remains an issue of clinical equipoise. The role of regular CAD screening is currently being assessed by the much anticipated Canadian-Australasian Randomised Trial of Screening Kidney Transplant Candidates for Coronary Artery Disease (CARSK; ClinicalTrials.gov NCT036743307) which will randomise patients to either no further screening after wait-listing or regular screening as per current practice. Preliminary modelling based upon the CARSK protocol has already demonstrated the potential cost effectiveness of a no further screening approach in Australia and New Zealand [301].

The absence of benefit from coronary revascularisation in chronic dialysis patients with stable CAD undermines the utility of screening and carries risk of potential harm as demonstrated by ISCHEMIA-CKD [287]. There is no evidence to support routine screening for CAD amongst asymptomatic dialysis patients not currently being considered suitable for transplantation. Active treatment of modifiable traditional risk factors, optimisation of dialysis therapy and active surveillance should remain the cornerstones of CAD management in these patients.

## 12. Conclusions & Future Directions

CAD remains an important cause of CVD in ESKD patients. The pathophysiology of CAD in ESKD is distinct from that in the general population because of the additive presence of non-traditional and uraemic specific CVD risk factors. Medical therapies for CAD appear to have a blunted efficacy in ESKD patients due in part to differences in disease biology and lack of high-quality randomised trials. Novel non-traditional risk factors such as chronic inflammation have recently been established as important therapeutic targets by the CANTOS trial and confirmed by other studies such as Low-Dose-Colchicine (LoDoCo) trials targeting the NLRP3 inflammasome pathway [302, 303]. Although the use of colchicine in ESKD is limited by potential gastrointestinal toxicity, other trial targeting inflammation such as ZEUS are currently underway in advanced CKD. The results of these will be instrumental for extending future trials to ESKD patients. Screening of stable CAD in asymptomatic ESKD patients on the transplant wait-list remains an important question which will be answered by the ongoing CARSK trial. The results of CARSK will have huge impact upon resource allocation, economic cost-to-benefit ratio and minimising dialysis wait-time. This is particularly relevant in the context of the recent ISCHEMIA-CKD trial suggesting that optimal medial therapy should be the favoured 1st line approach over invasive angiography and revascularisation for stable CAD in ESKD patients.

## Abbreviations

ACEi, angiotensin-converting enzyme inhibitor; ACR, albumin to creatinine ratio; ACS, acute coronary syndrome; AER, albumin excretion rate; AF, atrial fibrillation; AGEs, advanced glycation end-products; ANZDATA, Australia and New Zealand Dialysis and Transplant Registry; ARB, angiotensin II receptor blocker; AVF, arteriovenous fistula; BP, blood pressure; CABG, coronary artery bypass grafting; CAC, coronary artery calcification; CAD, coronary artery disease; CCF, congestive cardiac failure; CKD, chronic kidney disease; CKDEPI, CKD Epidemiology Collaboration; CTCA, computerised tomography coronary angiography; CVA, cerebrovascular accident; CVD, cardiovascular disease; DOPPS, Dialysis Outcomes and Practice Patterns Study; DSE, dobutamine stress echocardiogram; eGFR, estimated glomerular filtration rate; ERA-EDTA, European Renal Association-European Dialysis and Transplant Association; ESKD, end-stage kidney disease; EST, exercise stress testing; FFR, fractional flow reserve; GFR, glomerular filtration rate; HD, haemodialysis; HDL-C, high density lipoprotein cholesterol; hsCRP, highly sensitive C-reactive protein; iFR, instantaneous wave free ratio; IL, interleukin; IVUS, intravascular ultrasound; KDIGO, Kidney Disease: Improving Global Outcomes; KDOQI, National Kidney Foundation Kidney Disease Outcomes Quality Initiative; LDL-C, low density lipoprotein cholesterol; LVH, left ventricular hypertrophy; MACE, major adverse cardiovascular events; MDRD, Modification of Diet in Renal Disease; MI, myocardial infarction; MPS, myocardial perfusion scan; NHANES, National Health and Nutrition Examination Survey; NLRP3, nucleotide-binding leucine-rich repeat-containing pyrin receptor 3; NSTEACS, non-ST elevation acute coronary syndromes; NYHA, New York Heart Association; OCT, optical coherence tomography; PAD, peripheral arterial disease; PCI, percutaneous coronary intervention; PCSK9, proprotein convertase subtilisin/kexin type 9; PD, peritoneal dialysis; PE, pulmonary embolism; QCA, quantitative coronary angiography; RAAS, renin-angiotensin-aldosterone systome; SCD, sudden cardiac death; TIA, transient ischaemic attack; VHD, valvular heart disease; VTE, venous thromboembolism; SPECT, single-photon emission computerised tomography; STEMI, ST-elevation myocardial infarction; TG, triglycerides.
